# Editorial: From Thinker to Doer: Creativity, Innovation, Entrepreneurship, Maker, and Venture Capital

**DOI:** 10.3389/fpsyg.2021.649037

**Published:** 2021-03-08

**Authors:** Yenchun Jim Wu, Chih-Hung Yuan, Mu-Yen Chen

**Affiliations:** ^1^Graduate Institute of Global Business and Strategy, National Taiwan Normal University, Taipei, Taiwan; ^2^School of Economics and Commerce, University of Electronic Science and Technology of China, Zhongshan Institute, Zhongshan City, China; ^3^Department of Information Management, National Taichung University of Science and Technology, Taichung, Taiwan

**Keywords:** innovation, entrepreneurship, maker, venture capital, creativity

Entrepreneurship is a process that can be undertaken by individuals or groups of individuals. In their pursuit of entrepreneurial success, entrepreneurs are often under a relatively significant amount of psychological influence. Hence, the Research Topic not only explores the interaction between psychology and entrepreneurial ecosystem including entrepreneurship, maker, innovation, creativity, and venture capital, but also summarizes the latest developments and methodological contributions. This Research Topic spans a body of work that represents the effort of 53 academic papers from 198 authors. The following section will elaborate on entrepreneurship, maker, innovation, creativity, and venture capital, respectively.

## Entrepreneurship

Considering the advance law-based governance implemented in China, Miao examined the relationship between psychological understanding and the behavioral choices of entrepreneurs in the legal and artificial intelligence industries. The results found that the behavior choice of entrepreneurs in the legal industry are highly correlated with psychological understanding. The entrepreneurs' psychological understanding and behavior choice in the field of artificial intelligence do not show a direct correlation but indirect correlation through indirect factor innovation opportunities. After studying the impact of various psychological leadership styles of entrepreneurs on organizational learning abilities and performance, Tong found that the transformational psychological leadership style was positively correlated with organizational learning abilities, financial performance, and growth performance. Xu and Zhang conducted a questionnaire survey with 305 multinational entrepreneurs to understand the psychological effects of trade frictions. The results show that economic pressure was positively correlated with diminished thoughts and self-efficacy, and that diminished entrepreneurial thoughts caused by economic pressure could be reversed through social support.

Baluku et al. studied the impacts of positive psychological attributes on entrepreneurial intention and action. The results demonstrated that having firm implementation intentions is crucial to translating intentions into actions. Based on the personality development theory, Chen and Yu constructed a recommendation algorithm system for entrepreneurial projects under an optimized deep neural network. The research revealed that the optimized deep neural network could effectively enhance college students' entrepreneurial intentions and mental resilience. Chien-Chi et al. explored the relationship between emotional competence, entrepreneurial self-efficacy, and entrepreneurial intentions, discovering that entrepreneurial self-efficacy mediates the relationship between emotional competence, and entrepreneurial intentions.

Li H. et al. approached entrepreneurship education from the perspectives of psychology and literary ethics. The results show significant correlations among entrepreneurship education, entrepreneurial intentions, and entrepreneurial efficacy. Sun et al. investigated the interrelationships among four specific entrepreneurial characteristics (i.e., the need for achievement, locus of control, risk-taking propensity, and creativity) and their systematic influence on engineering students' entrepreneurial intentions. The study found that, while creativity and risk-taking propensity directly affect entrepreneurial intentions, the need for achievement and locus of control do so indirectly.

Wu W. H. et al. explored the cognitive processes underlying entrepreneurial intentions through entrepreneurial awareness, revealing that Taiwan's medical universities' entrepreneurship education did not align with the best-recommended practices. This was mainly because most teachers had little or no opportunity to practice or experience entrepreneurship. Yin et al. analyzed the relationships among college students' entrepreneurial psychological quality, psychological education, and entrepreneurial intentions. They identified inadequate entrepreneurial ability and the lack of professional knowledge as the leading causes of the students' negative attitudes toward entrepreneurship. While past studies on social entrepreneurial intentions mostly concentrated on the impacts of bright personalities, Wu W. et al. discussed the relationship between the dark triad and social entrepreneurial intentions by highlighting the mediating role of moral disengagement and the moderating role of empathic concern and perspective-taking. The dark triad negatively correlated with social entrepreneurial intentions mediated by moral disengagement. The results also indicated that education efforts might help mitigate the dark triad's negative impacts on social entrepreneurial intentions.

Du J. et al. delved into the impacts of different types of cultivation models in social entrepreneurship education on students' social entrepreneurship and concluded that the teacher-student co-entrepreneurship model is more effective and conducive to practical learning, compared to the traditional cultivation model. Qin et al. examined the effects of lean entrepreneurship education under the flipped classroom mode, indicating that incorporating the flipped classroom in the context of lean entrepreneurship effectively achieves the goal of deep learning ideally. Wang and Chiou shed light on the key factors influencing the willingness to implement online entrepreneurship programs (OEPs) among universities' Business Management departments, and the effectiveness of such OEPs. The results show that although innovative factors influence the departments' willingness to implement OEPs significantly, organizational, and environmental factors exert a greater influence on OEP implementation effectiveness.

Li Y. et al. probed the determinants influencing the effectiveness evaluation for entrepreneurship education in medical universities. They concluded that teacher-student collaboration is particularly useful in understanding the latest professional knowledge dynamics, enhancing scientific research capabilities, and improving innovation and entrepreneurship abilities. Applying positive emotions as the mediating variable, Zhang B. et al. investigated the relationship between college students' psychological capital and entrepreneurial learning engagement. The results suggested that positive emotions do not have any mediating effect while the latter is influenced by the former.

Wang and Huang conducted a series of face-to-face interviews to determine college student entrepreneurs' various support needs. The results indicated that while the needs during the preparation stage and startup stage of entrepreneurship were similar in many ways, the needs during the failure stage differed significantly from those in other stages. Wu and Mao investigated the impacts of the business environment on urban students' entrepreneurial motivations and found that the latter is significantly influenced by students' perceptions of the socio-economic conditions, education, and the financial and non-financial support available.

Mu et al. used entrepreneurial groups of college students as a springboard for discussing the entrepreneurial performance of startups founded by college students and identified significant correlations among entrepreneurship education, entrepreneurial self-efficacy, and entrepreneurial performance. The entrepreneurial self-efficacy of college students was found to have good explanatory power and played a mediating role. Feng and Chen explored the psychological and behavioral impacts of entrepreneurial passion on entrepreneurs. The study found that, while the direct effect of harmonious passion on entrepreneurial persistence and enterprise performance is insignificant, compulsive passion is significant. Zeng and Ouyang focused on the effects of tenacity and future self-continuity on inter-temporal risky choices under the entrepreneurial context and found that tenacity positively correlates with risky choices and inter-temporal risky choices, in which commitment, endurance, and challenge play a dominant role. Liu et al. investigated whether there was any price discrimination against the products offered by minority entrepreneurs among consumers. The findings suggested that potential consumers were biased against common products from minority enterprises but not products with ethnic characteristics.

## Creativity

Liang and Yuan investigated the relationships between creative self-efficacy, parenting style, the parent–child relationship, and after-school programs. Research has found that such programs possessed the most important variable affecting students' creative self-efficacies. The evidences also supported the view that negative parent–child relationships do not stimulate students' creative self-efficacies. After studying the relationship between creativity and entrepreneurial intentions, Shi et al. proposed that entrepreneurial education should focus on cultivating students' creativity and entrepreneurial efficacy. At the same time, their entrepreneurial intentions should be encouraged, and their entrepreneurial skills and thinking styles should be developed. Shu et al. examined creativity and innovation from the perspective of education and proposed a sustainability-oriented framework for creativity, innovation, and entrepreneurial education. They hoped that the proposed framework would transform thinkers into makers. In the study by Tien et al. the effects of different learning strategies used by student organizations on creativity were explored. The results reveal that in terms of creativity, groups that adopted the flipped classroom learning approach performed significantly better than groups that followed traditional learning strategies.

Gao Q. et al. conducted a survey on entrepreneurs' psychological capital, creative innovative behavior, and corporate performance, and found that the first two factors helped improve the third factor. Gao Y. et al. further pointed out that in the creative industries, entrepreneurs' creativity is the source of entrepreneurial activities. Their research found that entrepreneurial creativity positively affected individuals' propensity toward entrepreneurship, and that the relationship between neurotic and extroverted personalities and creativity was negative and U-shaped. Following a study on the relationship between workplace stress and employee creativity, He et al. found that the former was negatively correlated with the latter, and that it was mediated by creativity self-efficacy. Shen C. et al. explored how and when abusive supervision could damage employee creativity, and established that the former negatively affected the latter. In addition, the creative role identity mediated the relationship between employees' creativity and abusive supervision.

## Innovation

After exploring the effects of learning in a social networking environment on students' academic performance and learning behavior, Su and Chen concluded that academic performance and development of innovation were enhanced by it. This implied that a large cycle of social interactions and discussions existed. Zhang studied the teaching of innovation in higher education by combining the theory of humans' all-round development with deep learning. The results indicated that over-reliance on teachers greatly reduced students' preference for innovation and obliterated their innovative ideas. Zhang Q. et al. explored a model that could effectively improve college students' sense of responsibility regarding innovation and entrepreneurship, and which could facilitate success in their entrepreneurial endeavors. They concluded that positive psychological qualities—such as syngroup, excellence, modesty, benevolence, super-excellence, bravery, restraint, and wisdom—were the influencing factors for a sense of responsibility. After studying the effect that innovation education has on the collaboration and strategic innovations of students' entrepreneurial teams, Zhao et al. confirmed that a positive correlation existed between said education, teamwork, and strategic innovations.

Gao Q. et al. investigated the relationship between entrepreneurial psychological capital and employees' deviant innovative behaviors. The results demonstrated a significant correlation between entrepreneurial psychological capital and entrepreneurial performance. In addition, employees' “work values and psychological empowerment” were significantly related to deviant innovative behaviors. Through an analysis of the effect of ambidextrous learning on the performance of startups in ecological innovations, Huang et al. found that during the process of organizational learning of flexibility, startups should help raise their senior managers' environmental awareness to improve overall performance in ecological innovations. Li T. et al. analyzed ways of using leaders' psychological capital to improve employees' innovative behaviors. The results show that relationship exchanges between leaders and members, and the leaders' psychological capital had significant and positive impacts on employees' innovative behaviors. After examining the effects of psychological capital on deviant innovative behaviors, Xu and Zhao discovered that entrepreneurs' psychological capital plays a critical role in regulating work values and deviant innovative behaviors.

Chen J. et al. studied how coping combinations affected innovative abilities in cases of business failure. They pointed out that coping combinations could enhance flexibility in innovation through the reshaping of entrepreneurs' cognitive structures. Based on a study of the ways a dynamic team environment affected innovations by entrepreneurial teams, Deng et al. concluded that such environments stimulated innovations by triggering team members' agreement-seeking behavior, thereby facilitating the teams' knowledge integration. The research focus of Pan et al. was the relationship between the salary gaps and innovative efficiencies of enterprises. Their findings revealed that the internal salary gaps within the senior management team had a significant and positive impact on corporate innovative efficiencies. Wei et al. discussed the ways in which entrepreneurial self-efficacy (ESE) affected innovative behavior. Their research findings show that ESE had a significant and positive impact on entrepreneurs' innovative behaviors, with job satisfaction playing a mediating role between ESE and innovative behaviors.

## Maker

During an AIoT Maker course, Chen S. Y. et al. applied head-mounted virtual reality technology to the teaching of computational thinking. Students considered different scenarios through computational thinking, and discussed each scenario to identify maker designs that are suitable for AIoT projects. Jeng et al. investigated the effects of maker education on students' attitudes toward computational thinking. Research shows that maker education has a positive effect on students' abilities to master computational skills. Sun established a more systematic plan for implementing innovation and entrepreneurship education in colleges by combining Internet information technology and maker education, while Yang investigated the development of maker education in higher education and discovered that the development of maker activities cultivated positive psychological qualities in students during their entrepreneurial endeavors.

## Venture Capital

After examining the effects of customers' psychological prices on audit pricing, Ding found that a position of product market dominance reduced the agency costs between the owners and managers of the client's company. Du P. et al. revealed the relationship between information asymmetry and investment rate, as well as the critical need to analyze, understand, and seize the market. They also found that information asymmetry between the actual environment and the investment environment was more serious than previously thought. The role of venture capital (VC) in the process of transforming the innovation capabilities of enterprises into benefits was explored by Jin et al. Their study was based on the heterogeneity of human capital, and they established that the educational level of entrepreneurs' VC human capital and the proportion of engineering professionals effectively enhanced innovative capabilities of enterprise.

The focus of the study by Shen T. et al. was the ways in which major investors psychologically influenced follower investors, thereby affecting fundraising performance during equity crowdfunding. The results show a positive correlation between financing performance and the proportion of the financing target constituted by the investments of major investors, and their investment experiences. According to Xie et al. entrepreneurs could use vicarious learning to overcome the external uncertainties of overseas investments. After entrepreneurs gained foreign experiences from their interlocking partners, they could significantly promote the international growth of their companies when investing in the same country. After analyzing the effects of VC on corporate innovative activities, Zhang C. et al. discovered that the involvement of VC positively affected the innovative inputs and outputs of enterprises. Zhong et al. analyzed the pricing mechanism of initial public offerings and investigated the influence of investors' psychology on the pricing. Their conclusion was that investors' sentiments reflected their psychological states at that time, causing earnings to increase or decrease under the influence of investors' and market sentiments.

The objective of the Research Topic is to examine the activities involved at different stages during the entrepreneurial process from the psychological perspective. Hopefully, it can initiate an interdisciplinary scientific research dialogue between entrepreneurship and psychology. In conclusion, the Research Topic includes 53 research papers from different disciplines which may potentially provide researchers and practitioners with diverse views in their search for competitive solutions. These papers provide valuable ideas, strategies and activities for researchers and practitioners to effectively solve their problems in the field. Finally, many thanks go to the authors and reviewers who made contribution to the Research Topic for their time and effort.

## Author Contributions

YJW conceived of the idea and coordinated the Research Topic. C-HY and M-YC carried out support tasks for the coordination of the Research Topic and Edition of Articles. All authors contributed to the article and approved the submitted version.

## Conflict of Interest

The authors declare that the research was conducted in the absence of any commercial or financial relationships that could be construed as a potential conflict of interest.

